# Exploring the relationship between media use and depressive symptoms among gender diverse youth: findings of the Mental Health Days Study

**DOI:** 10.1186/s13034-024-00797-x

**Published:** 2024-08-22

**Authors:** Diana Klinger, Paul L. Plener, Golli Marboe, Andreas Karwautz, Oswald D. Kothgassner, Tobias Dienlin

**Affiliations:** 1 Department of Child and Adolescent Psychiatry, Medical University of Vienna, Vienna, Austria; 2 Comprehensive Center for Pediatrics, Medical University of Vienna, Vienna, Austria; 3 Department of Child and Adolescent Psychiatry and Psychotherapy, University of Ulm, Ulm, Germany; 4 Association for the Support of Self-determined Use of Media (VsUM), Vienna, Austria; 5 Department of Communication, University of Vienna, Vienna, Austria

**Keywords:** Gender diverse, Media use, Depressive symptoms, Adolescents, Youth

## Abstract

**Background:**

Over the past decades, media use has become a key aspect of young people’s daily lives, significantly shaping their social interactions, learning processes, and recreational pursuits. At the same time, healthcare professionals and researchers are increasingly concerned about the impact of media use on young people’s mental health. This concern is particularly relevant for gender diverse youth who may have distinct experiences with media that could impact their mental health uniquely compared to their peers, such as increased exposure to cyberbullying and negative content regarding their gender identity. This study aims to explore the associations between media use and depressive symptoms among youth and examine if gender moderates this association.

**Methods:**

This study utilized a cross-sectional design involving a school-based sample of 8158 participants (*M*_age_ = 14.05 years, *SD* = 2.45, *N*_diverse_ = 144) from Austria. Participants completed a survey assessing their media use and depressive symptoms using the Patient Health Questionnaire-9 (PHQ-9). Media use was measured by asking participants to report their daily usage in hours and minutes across various categories, including smartphone use, streaming services, social networks, and other media types. Multiple regression analyses were conducted to examine relationships between different forms of media use and depressive symptoms. Moderation analyses were performed using the PROCESS macro for SPSS to explore the role of gender.

**Results:**

For gender diverse youth, multiple regression analysis identified streaming services (β = 0.265, *p* = .005) and social networks (β = 0.189, *p* = .037) as significant predictors of depressive symptoms in gender diverse youth. Moderation analyses conducted with the entire sample showed that gender moderates the relationship between depressive symptoms and smartphone use (*B* = - 0.008, *p* = .014), with the effect being the most negative for gender diverse individuals.

**Conclusion:**

The findings underscore the complex relationship between media use and depressive symptoms among gender diverse youth, emphasizing the moderating role of gender. These results underline the need for gender-sensitive approaches in media literacy and mental health interventions. Stakeholders should be aware of risks and benefits of different media types to foster healthy media engagement.

**Supplementary Information:**

The online version contains supplementary material available at 10.1186/s13034-024-00797-x.

## Background

In recent years, media use has become an integral part of the daily life of youth worldwide, significantly influencing their social interactions, knowledge acquisition and leisure activities [1–3]. Concurrently, there has been a growing interest among mental health professionals and researchers about the potential impact of media use on the mental health of youth [4–9]. This interest is particularly pronounced for gender diverse youth, who may experience unique interactions with media that can affect their mental health differently from their peers [10].

Media use refers to the interaction and consumption of various forms of media, including activities like watching television or online videos, playing video games, reading books and newspapers, using social media, browsing websites and engaging with multimedia content on various devices such as smartphones, computers, tablets and other platforms [11, 12]. The use of media among adolescents and young adults has significantly increased worldwide, along with the widespread availability of digital technologies and online platforms. In the United States, research has shown that young people are spending more time on social media, streaming services and engaging with various interactive media platforms, surpassing the screen time of previous generations [13]. Similarly, in Germany, according to findings from the JIM study, young people’s digital engagement has expanded significantly. This includes a rise in smartphone usage throughout the 2010s, with smartphones becoming a central part of teenagers’ lives by the mid-2010s. Tablet usage also increased significantly, particularly during the COVID-19 pandemic, with a broad array of devices now integral to their daily activities. The results of the study indicate that nearly all households have access to these technologies, with smartphones, laptops, and televisions being most commonly owned by the youth themselves. This persistent access to digital devices has facilitated an increase in online activities, from video and music streaming to interactive gaming and social media use. On average, teenagers who use the function to track their screen time reported approximately 3.5 h of screen time per day on their smartphones. Additionally, the usage of streaming services has increased, with 86% of teenagers regularly using at least one streaming service. Furthermore, the study highlights gender differences in media use: girls are more likely to read books, use tablets, and listen to the radio, while boys are more likely to watch online videos and play digital games [14]. In Austria, a recent study has shown a diverse spectrum of media consumption among young people, indicating similar patterns in digital engagement. Smartphones remain the primary device for accessing a variety of digital media, including social networks, streaming services, and communication platforms like WhatsApp. Despite a general decrease in computer usage since the COVID-19 pandemic, specific activities like video gaming and streaming continue to be prevalent. Simultaneously, traditional media such as television maintain a consistent presence in daily media consumption and reading printed books continues to be a popular activity among the youth [15].

Building on this trend, specific patterns of media engagement have raised concerns regarding their potential impact on mental health, particularly in terms of how they may contribute to or exacerbate depressive symptoms. Previous studies suggest a connection between both problematic and general forms of media use and depressive symptoms [16], with problematic use specifically defined as the excessive engagement with media that disrupts daily functioning and leads to negative consequences in various contexts, such as social and academic settings [17]. Significant associations have been specifically found regarding excessive internet use [18], problematic internet use (PIU) and smartphone use (PSU) [19, 20], as well as video gaming [7, 21]. Numerous studies have specifically examined the relationship between depressive symptoms and both general social media use and problematic social media use (PSMU), including the use of social network sites, often indicating a significant influence [4–8, 21–23]. This is especially relevant given the prevalence of depression and elevated depressive symptoms among youth [24], which can significantly impact their development, self-esteem, and overall quality of life. At the same time, literature overviews also suggest positive relations between digital technology use and specific measures of well-being, including increased social support or increased social capital [16]. In addition, it is still unclear if the relationship is indeed causal [25], or if the effect sizes found are indeed large enough to be considered relevant [9, 26, 27]. In conclusion, given the widespread use of digital devices and the increasing amount of time young people spend online, understanding the relationship between different forms of media use and depressive symptoms is crucial for developing effective interventions and supporting their mental health.

Gender diverse is an umbrella term referring to individuals who identify with a gender other than their binary, birth-assigned sex and/or show consistent gender nonconformity. This term encompasses a variety of culturally diverse identities such as nonbinary, gender expansive, and gender nonconforming, as well as (binary) transgender individuals, distinctly different from cisgender individuals who identify with their sex assigned at birth [28, 29]. Previous studies have shown specific patterns of media use among transgender and gender diverse (TGD) youth compared to other LGBTQ+ subgroups and the general population. TGD youth, for instance, spend significantly more time online daily than their cisgender peers [30], with substantial internet and social media use, though slightly less on social media compared to the general population [10]. Additionally, youth with less traditional LGBTQ+ identities, such as pansexual, asexual, queer, and gender nonconforming, exhibit the highest use of mobile devices and online time [30].

Gender diverse individuals often encounter additional stressors due to their gender identity, beyond the general risks associated with media use, such as cyberbullying and discrimination [10, 31–34]. Although the broader narrative around youth media use typically emphasizes its adverse effects, the conversation shifts to a more nuanced perspective when considering LGBTQ+, including gender diverse youth, where significant positive influences, such as providing safe spaces and resources for identity exploration and community connection, have also been reported [10, 31–37]. Studies indicate that digital platforms like social media and online gaming environments serve as crucial venues for these youth to authentically express themselves, foster well-being, and receive peer support [31, 33, 36]. Particularly, the internet facilitates a range of supportive interactions from finding community and reducing stigma to enhancing self-expression and securing emotional and informational support [10, 35]. While quantitative research on this topic is more limited, it supports the findings of qualitative studies and reviews, showing that social media use is linked to better mental health outcomes and higher levels of personal satisfaction among LGBTQ+ youth [32, 37, 38]. This digital media use underscores the transformative potential of inclusive online spaces in supporting the mental health and developmental needs of marginalized groups [10, 31, 33–37, 39]. As research increasingly focuses on the mental health of gender diverse youth (including, e.g., binary transgender, non-binary, and gender nonconforming individuals), particularly their elevated risk of depressive symptoms compared to cisgender peers [40, 41], understanding how media use impacts these symptoms is crucial for addressing the disparities they face. In summary, despite growing evidence on the impact of media use on mental health, gaps remain in our understanding of how these effects vary among gender diverse youth. While prior studies have highlighted both positive and negative impacts, there is a need for more detailed exploration into how different forms of media influence depressive symptoms in this group. This study provides novel insights by focusing on gender diverse youth, an often marginalized population, and examining a wide range of media use types. These contributions aim to foster a better understanding of media’s impact on youth mental health. Therefore, this study aims to address following research questions:

1. How do gender diverse youth, in comparison to their peers with male and female gender, use different forms of media?
2. How is media use associated with depressive symptoms in gender diverse youth, considering different forms of media use?
3. Does gender serve as a moderating factor in the relationship between media use and depressive symptoms in youth?

Based on these research questions, we formulated the following hypotheses:

### H1

Gender diverse youth exhibit distinct media use patterns regarding the duration of usage (smartphone use, streaming, TV, social networks, messenger services, reading, news consumption, video games, and AI-based services) compared to their peers with male and female gender.

### H2

There is an association between the duration of media use and depressive symptoms in gender diverse youth, with variations depending on the type of media usage (streaming, TV, social networks, messenger services, reading, news consumption, video games, and AI-based services).

### H3

Gender moderates the relationship between media use and depressive symptoms, with the strength and direction of this relationship varying across gender groups.

**Fig. 1:**
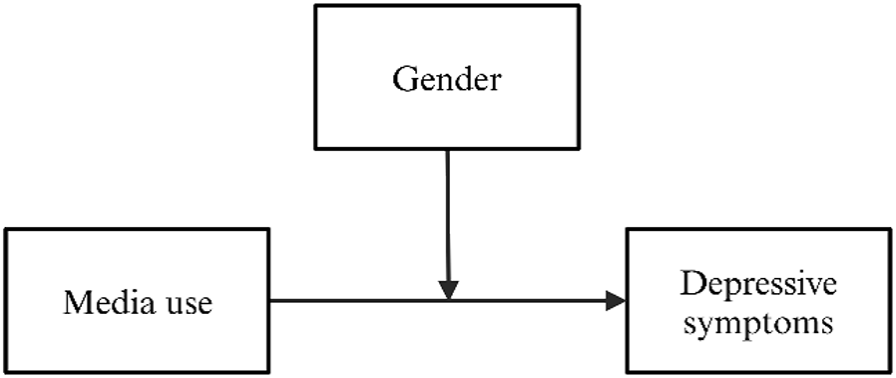
Conceptual diagram of the moderation model

To address the third research question and hypothesis, a moderation model is proposed where media use serves as the predictor of depressive symptoms with gender as moderator variable. This model aims to explore how the relationship between media use and depressive symptoms varies by gender (see Fig. 1 for a diagrammatic representation).

## Methods

### Participants and procedures

The study is being conducted in collaboration between the University of Vienna and the Association for the Support of Self-determined Use of Media (Verein zur Förderung eines selbstbestimmten Umgangs mit Medien; VsUM) as part of the Mental Health Days program. The program aims to educate students at Austrian secondary schools of various types (including grammar and vocational schools), starting from grade 5 (age 10 years and older), providing them with evidence-based practices and strategies for managing mental health challenges. The program involves the entire school community, including students, teachers, and guardians. Workshops tailored for different groups within the school are being conducted on the same day: students participate in a session equivalent to a school hour during regular class times, while separate events for teachers and guardians are held in the afternoon and evening, respectively.

The sample recruitment is being conducted through schools that expressed interest by completing an online form, after which they were systematically guided by the program’s team through the participation process. Data collection started in February 2023 and continues to date. This paper presents data from the initial year of the research (until February 2024). The data were collected via an online survey using the SoSci Survey platform [42], administered during the Mental Health Days event. Participants completed the survey in school after attending the workshops. The survey, which took approximately 20 min to complete, was administered either on mobile devices, tablets, or computers.

Participation in the study was voluntary, allowing students to withdraw at any time during the survey. Prior to the survey, detailed written information was provided to the legal guardians of students at participating schools, informing them about the Mental Health Days and the survey, and giving them the option to opt-out. Legal guardians were asked to provide passive consent on behalf of their children and were instructed to notify the teachers if they did not want their children to participate. To be included in the study, participants had to confirm their consent to participate and agree to the storage and use of their data in accordance with the General Data Protection Regulation (GDPR). At the beginning of the survey, participants were directed to the informed consent form and could only start the questionnaires after confirming their consent to participate. During completing the questionnaire, participants were supervised by trained teachers and social educators, as well as school psychologists, who were present to provide assistance if needed. All participants were debriefed and provided with explicit contact details and hotline numbers in case of problems. Approval for conducting the study and administering the survey was obtained from the Ethics Committee of the University of Vienna (no. 00887).

### Measures

#### Demographics

Among the demographic measures collected in the survey, the adolescents self-reported their age (in years) and gender. The question “What is your gender?” was used to assess gender, offering “female”, “male”, and “diverse/other” (hereafter referred to as gender diverse) as response options to cover a broader range of gender identities. For the original German version of the question, see Additional File 1. Participants could interpret and respond to the gender question based on their personal identification. This approach ensured inclusivity and aligned with the study’s objective to explore the experiences of gender diverse youth, which may include, among others, (binary) transgender, non-binary, and gender nonconforming youth, as well as individuals with differences in sex development.

#### Media use

Media use was assessed using self-constructed items that captured the duration and types of media consumed by participants. Participants were asked to report the average time they spent consuming various forms of media. This part of the survey initiated with a question asking, “How long do you typically spend per day with the following media?” This led to participants providing detailed information of their daily media usage across nine categories: smartphone use (in total), streaming online videos (e.g., YouTube, Twitch, Netflix), watching TV on the television set, using social networks (e.g., Instagram, TikTok, Facebook, Twitter), messaging apps (e.g., WhatsApp, Snapchat, Telegram, Signal), reading (books, e-books, magazines), news consumption (online or offline, image or text), playing video games (on mobile phones, PCs, or consoles) and using AI-based services (e.g., ChatGPT, DALL-E). Participants were instructed to record their usage in hours and minutes for each category, with an encouragement to use app-based tracking for accuracy if available. The questions and response options are detailed in Additional File 1, including the original German version and the English translation for reference.

#### Depressive symptoms

The German version of the Patient Health Questionnaire-9 (PHQ-9) was used to assess depressive symptoms [43]. The PHQ-9 is a self-report measure that evaluates the presence and severity of depressive symptoms over the last two weeks with nine items. Each item is scored on a Likert scale ranging from 0 (never) to 3 (nearly every day), with the sum score ranging from 0 to 27, where higher scores indicate greater symptom severity. Allgaier et al. [44] validated the scale for use in adolescent populations and reported good internal consistency and reliability (Cronbach’s *α* = 0.82).

### Data analysis

All statistical analyses were performed using IBM SPSS Statistics version 28 [45]. For continuous variables, means (*M*) and standard deviations (*SD*) were calculated. Categorical variables were summarized through frequencies and percentages. To compare media use across different genders, one-way ANOVAs were conducted for each media type. Due to unequal group sizes and violations of homogeneity of variances (except for watching TV), the Welch adjusted *F* ratio was used. A basic significance level of *p* <.05 was set for all comparisons. To address the issue of multiple comparisons, the Bonferroni method was applied, dividing the significance level (α) by the number of tests performed. With 10 different comparisons in our case, a corrected significance level of 0.05/10 = 0.005 was used when interpreting the data. Posteriori power calculation using G*Power [46] revealed a sufficiently powered sample size for multiple linear regression (*f*^2^=0.15, with 1-β = 0.90) and one-way ANOVA (*f* = 0.25 with 3 groups, 1-β = 0.99). Further, a multiple regression analysis was conducted to explore which forms of media use serve as significant predictors of depressive symptoms among gender diverse youth, using mean replacement for missing values. The overall percentage of missing data across all variables was 3.47%, with a range from 0.69% to 18.06%. All assumptions for the regression analysis were met, including the independence of distributions (Durbin–Watson statistic = 1.964) and no significant multicollinearity (VIFs ranged from 1.072 to 1.994). Total smartphone use was not included as a predictor due to its role as a platform facilitating access to various forms of media consumption such as streaming, social network use, and messaging services. Age was entered as the first block to control for its effects, followed by media use variables entered as a second block using the enter method. This strategy was chosen to provide insights into the specific media consumption behaviors that significantly predict depressive symptoms within this subgroup. Subsequently, moderation analyses were conducted using the significant predictors identified in the multiple regression analysis, as well as the total smartphone use, to further explore the moderating role of gender in the relationship between media usage and depressive symptoms. For the moderation analyses, the PROCESS Macro for SPSS, version 4.2 [47], was employed using data of the participants from the sample who provided information on their gender, including male, female, and gender diverse adolescents. Gender was coded and included as a moderator, with gender diverse individuals serving as the reference category, to assess whether the impact of specific forms of media use on depressive symptoms varies across genders. Each media use variable was analyzed separately to clearly identify the unique interaction effects of each media type with gender on depressive symptoms. Age was included as a covariate in all analyses to adjust for its potential impact on the results. For all moderation analyses, a 95% confidence interval with 5,000 bootstrapping samples was employed, while continuous variables were mean-centered. In the moderation analyses, a *p*-value of less than 0.05 was considered statistically significant.

## Results

### Demographic characteristics

A total of 9860 participants initiated the online survey, with 8654 completing it, resulting in an 87.8% completion rate. Furthermore, 496 respondents were excluded from the analysis as part of data cleaning measures, which included identifying and removing responses considered mischievous or unreliable, such as implausible age entries, school grade information, or unrealistic media usage times. The final sample consisted of 8158 participants aged between 10 and 24 years, with a mean age of 14.05 years (*SD* = 2.45). Out of the analytic sample, 452 students (5.5%) did not report their gender. Among those who reported their gender, *n* = 4421 participants were female (57.4%), *n* = 3141 were male (40.8%) and *n* = 144 identified as gender diverse (1.9%).

### Media use by gender

Table 1 summarizes the media use patterns across gender groups, highlighting significant differences in usage. Overall, gender diverse individuals reported the highest average use of several media types, particularly in smartphone use, streaming, and video games. Smartphone use was the highest among gender diverse individuals with a mean of approximately 4.73 h per day, compared to peers with female (3.96 h) and male gender (3.33 h). The results of the ANOVA indicated a significant difference across genders (*F*(2, 378) = 75.49, *p* < .001, η² = 0.02). Video games showed the most substantial difference, with gender diverse youth spending approximately 2.24 h per day, far exceeding the usage by youth with female (0.57 h) and male gender (1.96 h). This difference was highly significant (*F*(2, 370) = 608.11, *p* < .001, η² = 0.16). Similarly, streaming was notably higher in gender diverse youth, averaging about 2.19 h per day, compared to participants with female (1.45 h) or male genders (1.69 h). This difference was statistically significant (*F*(2, 372) = 30.41, *p* < .001, η² = 0.01). In addition to these, significant differences were found in other media types such as social networks, messenger services, reading, and AI, with gender diverse individuals consistently showing higher usage. Notably, TV watching did not differ significantly across gender groups (*F*(2, 379) = 1.52, *p* = .220, η² = 0.00). These results highlight the varying media consumption patterns among different gender groups, with gender diverse youth exhibiting significantly higher engagement across most media types.

**Table 1:** Means, standard deviations, and one-way analysis of variance of media use by gender

Media use type	Gender												
	Gender diverse		Female		Male		Total		ANOVA				
	*M*	*SD*	*M*	*SD*	*M*	*SD*	*M*	*SD*	*F*	*df1*	*df2*	*p*	η^2^
Smartphone use	283.65	168.25	237.77	147.83	199.97	135.92	223.20	144.91	75.49	2	378	< 0.001	0.02
Streaming	131.63	119.01	87.02	86.71	101.66	96.46	93.81	91.89	30.41	2	372	< 0.001	0.01
TV	42.23	69.66	44.62	62.61	41.99	66.75	43.51	64.46	1.52	2	379	0.220	0.00
Social networks	129.44	119.63	104.98	110.53	78.21	95.16	94.55	105.64	69.80	2	381	< 0.001	0.02
Messenger services	92.96	137.31	73.94	82.36	49.87	70.32	64.49	80.05	96.18	2	373	< 0.001	0.02
Reading	47.94	83.58	44.65	72.81	29.50	57.94	38.54	67.79	51.13	2	371	< 0.001	0.01
News	41.38	95.76	25.24	59.52	21.93	58.05	24.19	59.85	5.21	2	368	0.006	0.00
Video games	134.69	164.82	34.02	68.66	117.46	122.62	70.03	105.32	608.11	2	370	< 0.001	0.16
AI	24.86	74.31	7.24	31.30	11.19	39.17	9.20	36.11	11.20	2	306	< 0.001	0.01

Media use is reported in minutes per day

*ANOVA* = analysis of variance

### Depressive symptoms by gender

The results in Table 2 indicate significant differences in depressive symptoms among gender diverse, female, and male participants. Gender diverse individuals reported the highest mean PHQ-9 total score, indicating higher levels of depressive symptoms compared to participants with female and male genders. The ANOVA results revealed a statistically significant difference in depressive symptoms across the gender groups, *F*(2, 379) = 233.47, *p* < .001, with a medium effect size (η² = 0.06).

**Table 2:** Means, standard deviations, and one-way analysis of variance of depressive symptoms by gender

Depressive symptoms	Gender												
	Gender diverse		Female		Male		Total		ANOVA				
	*M*	*SD*	*M*	*SD*	*M*	*SD*	*M*	*SD*	*F*	*df1*	*df2*	*p*	η^2^
PHQ-9 total score	12.06	6.98	8.95	5.66	6.49	4.79	8.01	5.51	233.47	2	379	< 0.001	0.06

*ANOVA* = analysis of variance

### Multiple linear regression analysis: predictors of depressive symptoms in gender diverse youth

A multiple regression analysis, detailed in Table 3, was conducted to investigate the predictors of depressive symptoms as indicated by the PHQ-9 total score among gender diverse youth. In Model 1, age was entered first to assess its effect and was found not to be a significant predictor of depressive symptoms (β = 0.109, *p* = .194). Model 2 introduced the media usage variables – specifically, the use of streaming services, social networks, TV viewing, messenger services, reading, news consumption, video gaming, and AI services. This model, which accounted for age, explained 13.5% of the variance in PHQ-9 scores, representing an additional 13% variance explained compared to the 0.5% explained by Model 1, based on the adjusted *R*^2^ values. Within this model, the use of streaming services (β = 0.265, *p* = .005) and social networks (β = 0.189, *p* = .037) emerged as significant positive predictors of depressive symptoms, implying that increased usage of these forms of media is associated with higher depressive symptomatology. Conversely, TV viewing was a significant negative predictor (β = − 0.172, *p* = .044), suggesting that higher levels of TV consumption may be associated with lower levels of depressive symptoms. It should be noted that the effects of social networking sites and TV were only marginally significant. When examining effect sizes, the results suggest that these effects are small-to-medium-sized, based on Cohen’s criteria which classify effect sizes as small (β = 0.1), medium (β = 0.3), and large (β = 0.5) [48]. The remaining forms of media use, such as messenger services, reading, news consumption, video gaming, and AI services, did not significantly predict depressive symptoms in the gender diverse subsample.

**Table 3:** Results of multiple linear regression analysis predicting depressive symptoms in gender diverse youth

Predictor	*B*	*SE*	β	*t*	*p*
Model 1					
Constant	6.894	3.994		1.726	0.086
Age	0.342	0.262	0.109	1.306	0.194
Model 2					
Constant	7.592	3.902		1.946	0.054
Age	0.094	0.257	0.030	0.364	0.716
Streaming	0.016	0.006	0.265	2.827	0.005
TV	− 0.017	0.009	− 0.172	− 2.036	0.044
Social networks	0.011	0.005	0.189	2.110	0.037
Messenger	− 0.002	0.004	− 0.030	− 0.340	0.734
Reading	0.010	0.007	0.114	1.416	0.159
News	0.010	0.008	0.137	1.268	0.207
Video games	− 0.005	0.004	− 0.118	− 1.180	0.240
AI	0.009	0.011	0.089	0.810	0.420

Model 1: *R*^2^ = 0.012, adjusted *R*^2^ = 0.005, *F* = 1.707, *p* =.194; Model 2: *R*^2^ = 0.189, adjusted *R*^2^ = 0.135, *F* = 3.475, *p* <.001

### Moderation of the relationship between media use and depressive symptoms with gender as moderator variable

**Fig. 2:**
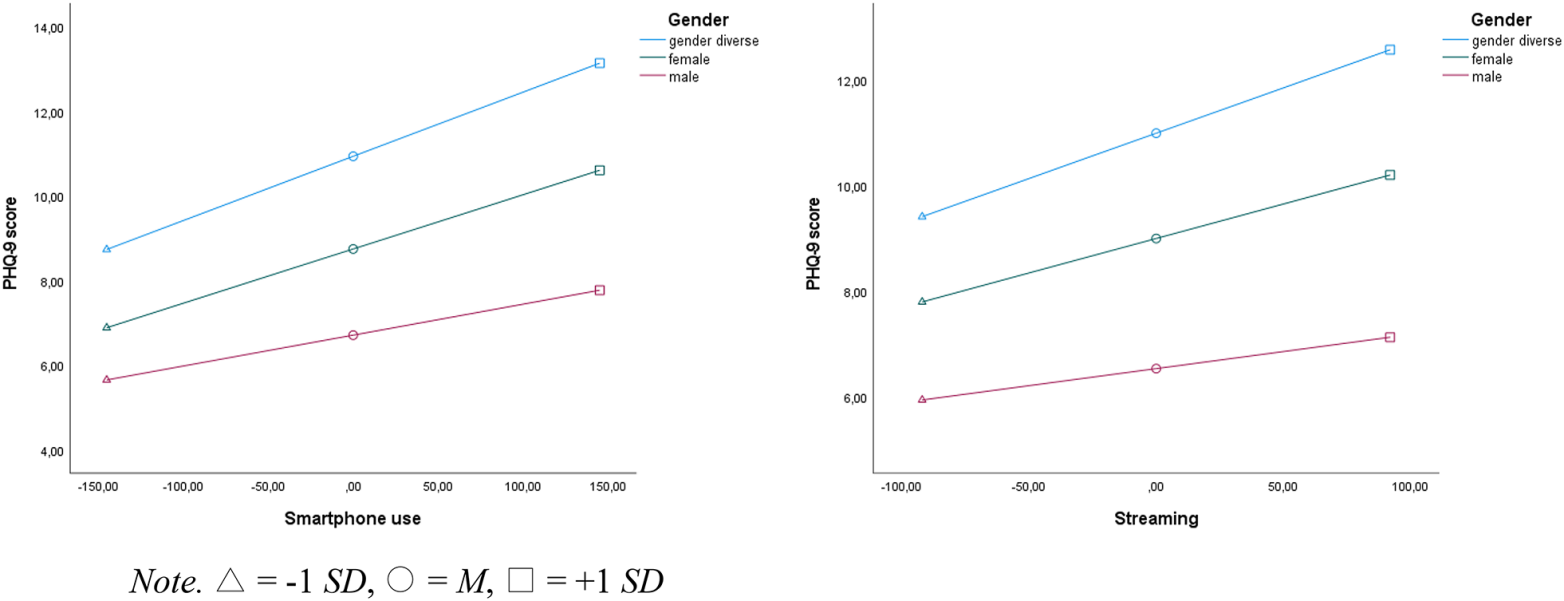
Results of the simple slope analyses of media use on depressive symptoms by gender

The results of the moderation analyses can be found in Tables 4, 5, 6 and 7, with detailed simple slopes analyses for significant interactions between media use and gender, presented in Fig. 2. The first moderation analysis, presented in Table 4, was performed to assess the interaction between smartphone usage and gender in predicting depressive symptoms, as measured by the PHQ-9 score. The overall model was significant, explaining 16.1% of the variance in depressive symptoms. Smartphone usage alone was found to be a significant predictor of depressive symptoms for gender diverse users, indicating that increased smartphone use is associated with higher levels of depressive symptoms. Furthermore, the conditional effects of gender were notably different for females and males, indicating that female and male participants had, on average, lower depressive symptoms compared to gender diverse individuals. Gender was found to moderate the relationship between smartphone usage and depressive symptoms. Specifically, the interaction term between smartphone usage and being male was significant, suggesting that for male participants the increase in depressive symptoms associated with higher smartphone usage is less pronounced compared to gender diverse individuals. The analysis of the conditional effects demonstrated that the influence of smartphone usage on depressive symptoms varied by gender: the effect was strongest for the reference group (gender diverse, *B* = 0.015), lessened for females (*B* = 0.013), and was weakest for males (*B* = 0.007). All effects were found to be statistically significant (*p* < .001).

**Table 4:** Results of moderation analyses regarding smartphone use and depressive symptoms with gender as a moderator variable

Effect	*B*	*SE*	95% CI		*t*	*p*
			*LL*	*UL*		
Predictor: smartphone use						
Constant	8.878	0.697	7.511	10.244	12.737	0.000
Smartphone use	0.015	0.003	0.009	0.021	4.872	0.000
Female gender	− 2.191	0.584	− 3.336	− 1.046	− 3.751	0.000
Male gender	− 4.226	0.586	− 5.374	− 3.078	− 7.218	0.000
Smartphone use x female gender	− 0.002	0.003	− 0.009	0.004	− 0.743	0.458
Smartphone use x male gender	− 0.008	0.003	− 0.014	− 0.002	− 2.467	0.014

Model smartphone use: *R*^2^ = 0.161; *F*(6, 7577) = 211.253, *p* < .0001. Additional variance explained by smart phone use and gender interactions: Δ*R*^2^ = 0.005; *F*(2, 7577) = 19.396, *p* < .0001.

*CI* = confidence interval; *LL* = lower limit; *UL* = upper limit

The second moderation analysis, shown in Table 5, was conducted to assess the interaction between the time spent with streaming online videos and gender on depressive symptoms. The overall model was found to be statistically significant, accounting for 11.9% of the variance in depressive symptoms. Results indicated that the time spent with streaming was a significant predictor of depressive symptoms for gender diverse users, showing that more time spent streaming correlates with higher depressive symptom levels. In terms of gender, the analysis showed significant conditional effects: females exhibited lower levels of depressive symptoms, and males demonstrated even lower levels, compared to the gender diverse group. Gender was found to significantly moderate this relationship, with the interaction term for streaming time and male gender being significant, pointing to gender differences in how streaming time affects depressive symptoms. In analyzing the conditional effects across gender groups, the influence of streaming time on depressive symptoms was strongest for the gender diverse group (*B* = 0.017), reduced for females (*B* = 0.013), and lowest for males (*B* = 0.006), with all effects significant at *p* < .001.

**Table 5:** Results of moderation analyses regarding the use of streaming services and depressive symptoms with gender as a moderator variable

Effect	*B*	*SE*	95% CI		*t*	*p*
			*LL*	*UL*		
Predictor: streaming						
Constant	6.103	0.715	4.703	7.504	8.541	0.000
Streaming	0.017	0.005	0.007	0.028	3.201	0.001
Female gender	− 1.990	0.618	− 3.203	− 0.778	− 3.218	0.001
Male gender	− 4.450	0.619	− 5.664	− 3.237	− 7.188	0.000
Streaming x female gender	− 0.004	0.005	− 0.015	0.007	− 0.750	0.453
Streaming x male gender	− 0.011	0.005	− 0.021	0.000	− 1.957	0.050

Model streaming: *R*^2^ = 0.119; *F*(6, 7565) = 156.971, *p* < .0001. Additional variance explained by streaming and gender interactions: Δ*R*^2^ = 0.003; *F*(2, 7565) = 10.158, *p* < .0001.

CI = confidence interval; *LL* = lower limit; *UL* = upper limit

In the next moderation analysis, the focus was on exploring the interaction between time spent with watching TV and gender as predictors of depressive symptoms (see Table 6). The model achieved statistical significance, explaining 8.8% of the variance in depressive symptoms. The analysis revealed that TV watching time by itself did not significantly predict depressive symptoms, suggesting no direct association between the amount of time spent watching TV and the level of depressive symptoms. In contrast, gender significantly influenced depressive symptoms, with decreases observed for female and male participants, compared to the gender diverse group, indicating conditional effects of gender. However, the interaction terms between TV watching time and gender for both female and male participants were not significant, indicating that gender does not significantly alter the relationship between TV watching time and depressive symptoms. The conditional effects analysis, aimed at evaluating the predictor’s influence at different levels of the moderator, showed no significant variation in the effect of TV watching time on depressive symptoms across gender categories.

**Table 6:** Results of moderation analyses regarding watching TV and depressive symptoms with gender as a moderator variable

Effect	*B*	*SE*	95% CI		*t*	*p*
			*LL*	*UL*		
Predictor: TV						
Constant	5.948	0.726	4.524	7.372	8.188	0.000
TV	− 0.001	0.020	− 0.039	0.037	− 0.045	0.964
Female gender	− 2.731	0.630	− 3.967	− 1.495	− 4.332	0.000
Male gender	− 5.022	0.631	− 6.259	− 3.784	− 7.955	0.000
TV x female gender	0.003	0.020	− 0.036	0.041	0.130	0.897
TV x male gender	0.004	0.020	− 0.035	0.042	0.181	0.856

Model TV: *R*^2^ = 0.088; *F*(6, 7545) = 126.617, *p* < .0001. Additional variance explained by TV and gender interactions: Δ*R*^2^ = 0.000; *F*(2, 7545) = 0.127, *p* = .881.

CI = confidence interval; *LL* = lower limit; *UL* = upper limit

Finally, a moderation analysis was performed to examine the effect of social network usage on depressive symptoms, exploring gender as a moderating factor, as detailed in Table 7. The analysis revealed a significant overall model, accounting for 13.7% of the variance in depressive symptoms. Social network usage emerged as a significant predictor, suggesting an association between higher usage of social networks and increased depressive symptoms in gender diverse youth. The interaction terms did not reach statistical significance for females and were also not significant for males, indicating that the effect of social network usage on depressive symptoms was not significantly moderated by gender. Analysis of the conditional effects further clarified these relationships, demonstrating that the influence of social network usage on depressive symptoms was most pronounced among the gender diverse group (*B* = 0.016), slightly reduced for females (*B* = 0.014), and least for males (*B* = 0.008), although the interactions did not reach the level of statistical significance.

**Table 7:** Results of moderation analyses regarding the use of social networks and depressive symptoms with gender as a moderator variable

Effect	*B*	*SE*	95% CI		*t*	*p*
			*LL*	*UL*		
Predictor: social networks						
Constant	7.745	0.714	6.345	9.145	10.847	0.000
Social networks	0.016	0.005	0.006	0.026	3.059	0.002
Female gender	− 2.395	0.606	− 3.584	− 1.207	− 3.951	0.000
Male gender	− 4.501	0.608	− 5.692	− 3.310	− 7.406	0.000
Social networks x female gender	− 0.003	0.005	− 0.013	0.008	− 0.470	0.639
Social networks x male gender	− 0.008	0.005	− 0.019	0.002	− 1.544	0.123

Model social networks: *R*^2^ = 0.137; *F*(6, 7547) = 170.332, *p* < .0001. Additional variance explained by social networks and gender interactions: Δ*R*^2^ = 0.003; *F*(2, 7547) = 9.635, *p* < .0001.

CI =confidence interval; *LL = *lower limit; *UL* =upper limit

## Discussion

The current study explored the relationship between media use and depressive symptoms, focusing on gender diverse youth while also examining youth of male and female genders. This study aimed to fill the gap in understanding how different forms of media consumption impact the mental health of gender diverse youth, who may have unique experiences compared to their peers. Additionally, we evaluated whether gender moderates these relationships, providing insights into the potential differences in the psychological effects of media use among gender diverse youth and their peers with male or female genders.

Gender diverse individuals reported the highest average use of several media types, notably smartphone use (approximately 4.73 h per day), video games (around 2.24 h per day), and streaming (about 2.19 h per day), compared to their peers. These differences were statistically significant, indicating a distinct pattern of media consumption among gender diverse youth. This aligns with findings from the study by McInroy et al. [30], which noted that transgender youth spend significantly more time online daily than their cisgender peers, and youth with less traditional LGBTQ+ identities, such as pansexual, asexual, queer, and gender nonconforming, exhibit the highest use of mobile devices and online time. Similarly, Herrmann et al. [10] found that TGD adolescents spend substantial time on the internet and social media, though slightly less on social media compared to the general population. In contrast, our results indicate that gender diverse youth use social networks more extensively. This difference might be attributed to the increased availability of online resources and support groups tailored to gender diverse youth, coupled with their greater need for social connectivity and community, which they fulfill through extensive use of social networks. Furthermore, gender diverse youth exhibited the highest levels of depressive symptoms, with a mean PHQ-9 score indicating higher depressive symptoms than their peers. This aligns with previous research, suggesting that gender diverse youth face greater mental health challenges [40, 41].

Multiple regression analysis identified the use of streaming services and social networks as significant predictors of depressive symptoms, while watching television appeared to have a protective effect. Interestingly, TV might offer positive effects by providing relaxing content or fostering social interaction when watched with others, in contrast to the potentially stress-inducing effects of online media. This aligns with existing literature suggesting that social networks and streaming services can exacerbate feelings of isolation and inadequacy [32, 49–51], particularly among gender diverse youth who may rely more heavily on digital environments for social interaction and support [37]. The immersive and interactive nature of these platforms often exposes users to idealized images and experiences, leading to detrimental social comparisons and enhanced perceptions of social isolation [52]. However, it is important to note that the cross-sectional nature of this study precludes any determination of causality. It is possible that higher levels of depressive symptoms could lead to increased media use, particularly of more immersive or interactive types, as individuals may seek distraction or solace online [53]. Conversely, the negative content and social comparisons prevalent in these media types could exacerbate depressive symptoms, creating a cyclical effect [31, 32, 41]. Contrary to this, traditional television consumption was inversely related to depressive symptoms, suggesting a potentially less harmful, or even beneficial, role of non-interactive media in the lives of these youths [54, 55]. When evaluating effect sizes, the results indicate that the observed effects range from small to medium, suggesting that although these effects are not large, they are also not negligible. Moreover, other forms of media such as reading, video games, news consumption, messenger services, and interactions with artificial intelligence showed no significant relationship with depressive symptoms. This may reflect their varied roles in users’ lives, balancing between positive interactions and potential sources of stress. The neutral impact of these kinds of media usage could suggest their use as tools for entertainment and cognitive engagement without the risks associated with social media and news consumption, likely due to a lower information overload, which can contribute to depressive symptoms [56].

Moderation analyses revealed that gender moderates the relationship between smartphone use and depressive symptoms, with the effect being strongest for gender diverse individuals, lessened for females, and significantly weakest for males. This may suggest that male youth have different usage patterns or attitudes towards smartphones that influence their psychological impact less than in gender diverse individuals. The significant moderation effect found in the use of streaming services underlines the importance of gender as a factor that influences the psychological effects of media use. Alternatively, it is conceivable that gender diverse youth with pre-existing depressive symptoms might engage more intensively with media as a form of escapism or to find communities where they feel accepted [57]. However, no significant gender moderation was found regarding watching TV and the use of social networks in relation to depressive symptoms. Interestingly, our study did not find the positive effects of media use, such as those often cited in qualitative studies regarding the supportive and identity-affirming aspects of media use for gender diverse youth [35, 36], in relation to depressive symptoms [38]. This discrepancy may indicate that the negative impacts of high media consumption outweigh the potential benefits for this group. In summary, responses to media influences varied among gender diverse youth, underscoring the importance of recognizing and addressing the unique needs and experiences within this group.

This study adds to the growing body of knowledge on the interactions between media use, mental health, and gender diversity, emphasizing the need for ongoing analysis of how media consumption affects gender diverse individuals. Further studies should investigate the supportive aspects of media as a protective tool for at-risk youth, particularly through enhancing media literacy [22]. These insights are essential for designing effective mental health strategies that maximize media benefits while minimizing risks. Our findings demonstrate that the connection between media use and depressive symptoms is influenced by gender and media types. This highlights the necessity for customized prevention and intervention methods to mitigate the negative impacts of media on youth mental health. It is crucial to integrate gender considerations into strategies promoting healthy media habits. Educators, parents, and policymakers need to be aware of the varying risks and benefits associated with different media types and should foster healthy media engagement. Moreover, interventions should account for the diverse effects of media across different genders, encouraging practices that enhance resilience to the negative impacts of problematic media use [38].

Our study’s strengths include its focus on gender diverse youth, an often overlooked population, and its school-based design, which enhances the validity of the data compared to purely online studies as participation was restricted to students attending schools. The inclusion of a wide range of media types provides a better understanding of media use patterns. Additionally, the school-based setting is enhancing the reliability and applicability of the findings, as the controlled setting allows for systematic data collection. However, the study also has limitations. Firstly, the lack of assessment of sex assigned at birth may affect the understanding of the impact of gender identity on the studied relationships. The reliance on participants’ personal identification in response to the gender question may introduce variability in how gender is understood and reported, potentially influencing the interpretation of the results. Moreover, the cross-sectional design prevents drawing causal inferences. Longitudinal studies are needed to better understand the temporal dynamics between media use and depressive symptoms. The selection of schools was not representative, which may limit the generalizability of the findings. The study procedures only allowed participation of those being present in person, which omits youth who dropped out of school or have serious mental health issues from partaking in the study. Data collection occurred after the workshops, which may have sensitized participants and influenced their responses. The self-reported nature of media use and depressive symptoms could introduce bias, requiring more objective measures in future studies. Additionally, the models explained a limited portion of the variance in depressive symptoms, indicating that other important factors should be considered, such as the influence of peers and family, which are known strong predictors of mental health problems among gender diverse youth [58]. Moreover, other dimensions of identity, such as sexual orientation/identity, were not assessed, which could have provided additional insights.

## Conclusion

Our study enhances the understanding of the relationship between media use and depressive symptoms among gender diverse youth, highlighting the need for gender-sensitive approaches in media literacy and mental health interventions. The findings demonstrate that the type of media and gender significantly influence depressive symptoms, requiring differentiated intervention strategies. Recognizing these variations is crucial in developing effective mental health programs that mitigate the negative impacts of media use while promoting positive engagement for gender diverse youth. The school-based approach of our study strengthens the validity of these findings, offering a structured environment for data collection that enhances the reliability of the results. Future research should employ longitudinal designs to further explore these relationships and evaluate targeted interventions’ effectiveness in reducing harmful media use effects. Moreover, it is essential to integrate comprehensive media literacy programs that address the specific needs of gender diverse youth, fostering resilience and well-being in the digital age.

### Electronic supplementary material

Supplementary Material 1

## Data Availability

The datasets used and/or analyzed during the current study are available from the corresponding author on reasonable request.
