# Supplementary material for: Exploring the relationship between media use and depressive symptoms among gender diverse youth: findings of the Mental Health Days Study
Source: Child Adolesc Psychiatry Ment Health. 2024 Aug 22;18:104. doi: 10.1186/s13034-024-00797-x (PMC11342596; doi:10.1186/s13034-024-00797-x)
Supplement: Supplementary file 1 — Supplementary Material 1 [file 13034_2024_797_MOESM1_ESM.docx]

**Additional file 1**

**Supplementary Information**

**Exploring the Relationship Between Media Use and Depressive Symptoms Among Gender Diverse Youth: Findings of the Mental Health Days Study**

Diana Klinger, Paul L. Plener, Golli Marboe, Andreas Karwautz, Oswald D. Kothgassner, Tobias Dienlin

**Questions of the Mental Health Days Study regarding gender and media use**

**What is your gender?**

***Welches Geschlecht hast du?***

- female
- *weiblich*
- male
- *männlich*
- divers/other
- *divers/anders*

**How long do you typically spend per day with the following media?**
Please enter your answer in the respective fields.
Example: If you read for about 20 minutes a day, enter "0" in hours and "20" in minutes.
If you have an app that tracks your time, feel free to check it.

***Wie lange verbringst du am Tag üblicherweise mit folgenden Medien?*** *Gib bitte deine Antwort in die jeweiligen Felder ein.
Beispiel: Wenn du ca. 20 Minuten am Tag liest, gib "0" bei Stunden und "20" bei Minuten ein.
Falls du eine App hast, die deine Zeit misst, kannst du dies gerne nachschauen.*

1. **Smartphone (total)**
   ***Smartphone (insgesamt)***
   ______ Hours ______ Minutes
   *______ Stunden ______ Minuten*
2. **Online videos via YouTube, Twitch, Netflix or similar
   *Online Videos über YouTube, Twitch, Netflix oder ähnliches***
   ______ Hours ______ Minutes
   *______ Stunden ______ Minuten*
3. **Television on a TV set
   *Fernsehen am Fernsehgerät***
   ______ Hours ______ Minutes
   *______ Stunden ______ Minuten*
4. **Social networking sites such as Instagram, TikTok, Facebook or Twitter
   *Netzwerkseiten wie Instagram, TikTok, Facebook oder Twitter***______ Hours ______ Minutes
   *______ Stunden ______ Minuten*
5. **Messengers such as WhatsApp, Snapchat, Telegram or Signal
   *Messengern wie WhatsApp, Snapchat, Telegram oder Signal***
   ______ Hours ______ Minutes
   *______ Stunden ______ Minuten*
6. **Books, eBooks and magazines
   *Bücher, eBooks und Zeitschriften***
   ______ Hours ______ Minutes
   *______ Stunden ______ Minuten*
7. **News (online or offline, image or text)
   *Nachrichten (online oder offline, Bild oder Text)***
   ______ Hours ______ Minutes
   *______ Stunden ______ Minuten*
8. **Video games (on mobile phone, PC or console)
   *Videospiele (am Handy, PC oder Konsole)***
   ______ Hours ______ Minutes
   *______ Stunden ______ Minuten*
9. **Services and software based on artificial intelligence (ChatGPT, DALL-E, ...)
   *Dienste und Software basierend auf künstlicher Intelligenz (ChatGPT, DALL-E, …)***
   ______ Hours ______ Minutes
   *______ Stunden ______ Minuten*
